# Motor cortex hemodynamic response to goal-oriented and non-goal-oriented tasks in healthy subjects

**DOI:** 10.3389/fnins.2023.1202705

**Published:** 2023-07-19

**Authors:** Michele Lacerenza, Lorenzo Frabasile, Mauro Buttafava, Lorenzo Spinelli, Elisa Bassani, Francesco Micheloni, Caterina Amendola, Alessandro Torricelli, Davide Contini

**Affiliations:** ^1^Dipartimento di Fisica, Politecnico di Milano, Milano, Italy; ^2^PIONIRS s.r.l., Milano, Italy; ^3^Istituto di Fotonica e Nanotecnologie, Consiglio Nazionale delle Ricerche, Milano, Italy; ^4^Scuola Universitaria Professionale della Svizzera Italiana, Manno, Switzerland; ^5^Centro Studi Riabilitazione Neurologica, Neuropsicologia e Neuropsicoterapia, Milano, Italy

**Keywords:** motor disorders, functional near infrared spectroscopy, supplementary motor area, neuromotor rehabilitation, cerebral hemodynamic response function, non-goal-directed movements, goal-directed movement, motor cortex

## Abstract

**Background:**

Motor disorders are one of the world’s major scourges, and neuromotor rehabilitation is paramount for prevention and monitoring plans. In this scenario, exercises and motor tasks to be performed by patients are crucial to follow and assess treatments’ progression and efficacy. Nowadays, in clinical environments, quantitative assessment of motor cortex activities during task execution is rare, due to the bulkiness of instrumentation and the need for immobility during measurements [e.g., functional magnetic resonance imaging (MRI)]. Functional near-infrared spectroscopy (fNIRS) can contribute to a better understanding of how neuromotor processes work by measuring motor cortex activity non-invasively in freely moving subjects.

**Aim:**

Exploit fNIRS to measure functional activation of the motor cortex area during arm-raising actions.

**Design:**

All subjects performed three different upper limbs motor tasks: arm raising (non-goal-oriented), arm raising and grasping (goal oriented), and assisted arm raising (passive task). Each task was repeated ten times. The block design for each task was divided into 5 seconds of baseline, 5 seconds of activity, and 15 seconds of recovery.

**Population:**

Sixteen healthy subjects (11 males and 5 females) with an average (+/− standard deviation) of 37.9 (+/− 13.0) years old.

**Methods:**

Cerebral hemodynamic responses have been recorded in two locations, motor cortex (activation area) and prefrontal cortex (control location) exploiting commercial time-domain fNIRS devices. Haemodynamic signals were analyzed, separating the brain cortex hemodynamic response from extracerebral hemodynamic variations.

**Results:**

The hemodynamic response was recorded in the cortical motor area for goal-oriented and not-goaloriented tasks, while no response was noticed in the control location (prefrontal cortex position).

**Conclusions:**

This study provides a basis for canonical upper limb motor cortex activations that can be potentially compared to pathological cerebral responses in patients. It also highlights the potential use of TD-fNIRS to study goal-oriented versus non-goaloriented motor tasks. Impact: the findings of this study may have implications for clinical rehabilitation by providing a better understanding of the neural mechanisms underlying goal-oriented versus non-goal-oriented motor tasks. This may lead to more effective rehabilitation strategies for individuals with motor disorders and a more effective diagnosis of motor dysfunction supported by objective and quantitative neurophysiological readings.

## Introduction

Motor neuron diseases are a group of neurodegenerative disorders related to upper and lower motor neuron degradation that strongly affect the global population. The study by Logroscino et al. (2018) shows a high incidence of neuromotor diseases reaching around 5% prevalence in the 50 years old world population and overtaking 20% incidence for individuals older than 70. This study also shows a higher incidence in the male population compared to the female one.

It is of primary importance to tackle an early diagnosis and implement prevention and monitoring plans, considering such prevalence of functional neurological disorder, its disabling consequences, and its costly handling. After being marginalized in the late 20th century, there has been renewed interest in this field in the last decade (Perez et al., 2021) for both diagnosis and treatments in patients with motor functional disorders, including also functional limb weakness. The resurgence of interest in this field encouraged us to enter this sector to help catalyze diagnostic and therapeutic improvements.

In the neuro-motor rehabilitation field, the selection of exercises and motor tasks to be performed by the patients is of paramount importance. It has been studied in previous literature the effect of goal-oriented (or goal-directed) exercises in motor learning mechanisms. Goal-oriented tasks require the subject to perform a specific movement with the intention of achieving a certain result, while non-goal-oriented tasks require the subject to simply perform a movement without any specific outcome in mind. In contrast with interventions that focus on changing body functions with simple motion tasks, goal-oriented training with activity-based approach to therapy proved to give meaningful advantages (Mastos et al., 2007; Wevers et al., 2009).

During goal-oriented tasks, the pre-motor cortex and the parietal cortex, which are associated with attention, planning, and execution of the movement, tend to be more active (Rizzolatti et al., 1987, 2014). Additionally, brain regions associated with feedback, such as the anterior cingulate cortex and the supplementary motor area (SMA) (Diedrichsen et al., 2006; Yamagata et al., 2012) tend to be highly involved during goal-oriented tasks, allowing for adjustments to be made based on the outcome of the movement.

Non-goal-oriented tasks instead, tend to activate brain regions associated with movement execution and sensory processing, such as the primary motor cortex and the supplementary motor area. These regions are responsible for the planning and execution of the movement itself, rather than the goal that the movement is trying to achieve. Brain regions associated with attention and feedback are typically less active during non-goal-oriented tasks, as the focus is on the movement itself rather than on its outcome.

Goal-oriented therapy has emerged as a promising approach to neurorehabilitation that emphasizes the setting of specific, achievable goals in order to guide and tailor rehabilitation interventions. Goal-oriented movements appear to produce a better reaching performance than the same movements performed without a specific goal (Wu et al., 2000) and can improve patient motivation and engagement in rehabilitation, which can further improve outcomes. In the study of Nathan et al. (2012), to understand neural correlates of upper extremity task executions (functional vs. non-functional) and their influence on neuromotor control strategies, it is found that neuromotor strategy for functional goal-oriented movements is different from rhythmic movements such as finger tapping or non-functional movements, and this difference can be quantified and mapped using functional magnetic resonance imaging (fMRI). These results support the concept of using goal-oriented tasks in rehabilitation and therapy for restoration of a function, that is task and context specific. Importantly, by tailoring rehabilitation interventions to specific goals and capacities of the patient, goal-oriented therapy can lead to more efficient and effective neurorehabilitation.

Despite these interesting and meaningful results, most of the motor learning studies performed in humans presented in literature, lack of a quantitative assessment of the actual involvement of the motor cortex real-time during the task execution. This is mainly due to the techniques used to assess brain activations, often based on fMRI, electroencephalography (EEG) and positron emission tomography (PET), which are highly limited by their bulkiness, cost and sensitivity to motion artifacts. Being able to quantify the motor cortex involvement in motor exercises in goal-oriented tasks compared to non-goal-oriented tasks would strengthen the hypothesis of higher effectiveness of goal-oriented ones in motor rehabilitation, confirming the results of previous studies based on less objective evaluations such as goal attainment scaling.

The scientific community envisages functional near-infrared spectroscopy (fNIRS) technology as a promising tool for providing easy and non-invasive access to cerebral hemodynamics, which can be paramount in the process of diagnosing and monitoring motor dysfunctions strongly related to neuro-functional processes (Leff et al., 2011). fNIRS is a non-invasive neuroimaging technique that measures changes in the concentration of oxygenated (O_2_Hb) and deoxygenated (HHb) hemoglobin in the brain by means of infrared light which penetrate the skull reaching the brain cortex. It is used to study brain functions in both research and clinical settings.

Time-domain (TD)-fNIRS (Lange and Tachtsidis, 2019), which exploits laser pulses instead of continuous wave light, outperforms standard fNIRS approach (Ortega-Martinez et al., 2023) and can unlock the possibility of quantitatively measuring the hemodynamic response, thanks to the discrimination between cerebral and extra-cerebral signals, potentially offering the ability to assess progress of patient conditions with physiological indexes (i.e., features of the hemodynamic response function, HRF) when treated with specific therapies or rehabilitation programs.

TD-fNIRS has been already used to measure functional motor cortex activation in various motor task exercises (Re et al., 2013; Muthalib et al., 2015; Lacerenza et al., 2021), also comparing results with other techniques such as EEG and fMRI (Visani et al., 2015). As examples, the study conducted by Muthalib et al. (2015) explores the potential of TD-NIRS as a neuroimaging tool to observe distinctive patterns of neural activity associated with external stimuli, such as sensory inputs, compared to voluntary actions; research conducted by Lacerenza et al. (2021) investigates how hemodynamic activation linked to similar motor actions can be differentiated based on hemodynamic activity patterns. No previous TD-NIRS studies have been found investigating differences in motor cortex activation related to goal-oriented and non goal oriented tasks. Compared to previous works performed with fMRI in humans on this topic (Nathan et al., 2012), in which the hemodynamic response was reported as the average activation intensity resulting form multiple task executions in a defined period (e.g., 30 s), this study aims to recover the actual temporal hemodynamic response due to the functional activation, including both oxygenated and deoxygenated hemoglobin concentrations during tasks execution (<10 s time period).

In this work, the investigation has been focused on studying differences in brain activations during goal-oriented motor tasks versus non-goal-oriented motor tasks via TD-fNIRS on healthy subjects. Volunteers’ brain hemodynamics was recorded in real-time from two locations: prefrontal cortex and motor cortex, with two synchronized TD-fNIRS devices (NIRSBOX, PIONIRS s.r.l., Milano, Italy) and different upper limbs motor tasks have been studied. The protocol was divided into three tasks: arm raising (active movement not goal-oriented), arm raising combined with grasping of a small object (active, goal-oriented movement), and helped arm raising (passive movement).

The possibility of having quantitative information on the brain activation (not affected by extra cerebral hemodynamic variations) will enable to retrieve reliable brain hemodynamic responses from these three motor tasks in healthy subjects. This will help in setting the basis of canonical upper limb motor cortex activations that will be potentially compared to pathological cerebral responses in future patients.

## Methods

### Subjects

In this study, 16 healthy volunteers, have been enrolled (11 males and 5 females) with an average (±standard deviation) of 37.9 (±13.0) years old. All subjects included in these measurements cooperated voluntarily and previously provided written informed consent to the procedures of the study, which was conducted according to the guidelines of the Declaration of Helsinki and approved by the Ethics Committee of Politecnico di Milano.

### Protocol

**Figure 1:**
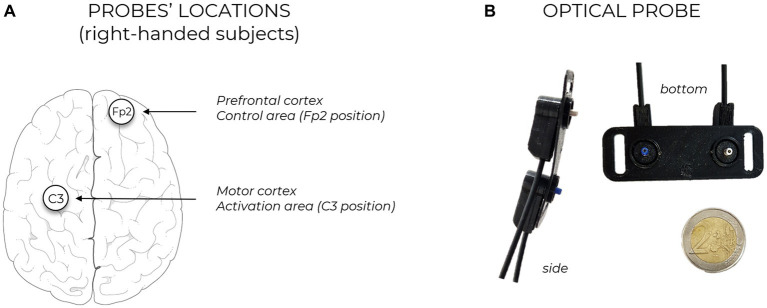
**(A)** Sketch of probe locations, in case of right-handed subject. The positioning of the probes is performed following EEG10/20 system mapping, injection and detection points are equidistant form the selected location. Each probe is connected to one TD-fNIRS device. The two devices are synchronized together for parallel and bilateral brain hemodynamic acquisition. **(B)** Pictures of the custom developed optical probe.

Protocol design and probe positioning were selected based on previous literature. To be able to record data from the motor cortex and the prefrontal cortex (as control location, where no significant brain activation is expected) two NIRSBOX devices (Lacerenza et al., 2022) (PIONIRS s.r.l., Milano, Italy) have been used. These instruments exploit two low-power (<5 mW) laser sources with wavelengths 685 nm and 830 nm; they acquire data at 1 Hz sampling frequency, transmitting to a laptop PC through USB connection and are synchronized together using hardware logic signals, to ensure true real-time acquisitions. The first unit was used to measure the region related to motor activation (C3 position following EEG10/20 system mapping, covering primary motor cortex and supplementary motor areas), while the second one was placed on the prefrontal region, Fp2 location (control channel). A schematic of the probe locations is reported in Figure 1A. Measurements have been performed in typical laboratory settings at Politecnico di Milano, a free area of at least six meters squared was left around the subjects to allow for unrestricted movement in space. Three left-handed subjects were present in the population under study and have been included in the analysis: in this case probe positions were C4 and Fp1, being motor task performed with the dominant arm. Unlike previous measurements and common fNIRS publications (in which subjects with dense or dark head hair are often excluded) in this work, we employed a dedicated optical probe design (PIONIRS s.r.l., Milano, Italy) which has allowed to recruit volunteers regardless of their scalp and hair characteristics. Source detector distance in this probe was 3 cm, with one injection and one detection channel. Retractable, spring-loaded optical fiber terminations were employed, impinging vertically on the head surface and facilitating direct contact with the skin without applying excessive pressure thus avoiding uncomfortable wearing by the subject. A picture of this probe is reported in Figure 1B. Probes have been secured with black elastic bandages around the head of the volunteers, without covering eyes and ears. For the simultaneous acquisition on the prefrontal cortex, a standard flat probe with horizontal-placed fiber cables has been used (B5 probe, PIONIRS S.r.l.) (PIONIRS S.r.l, 2023). Source/detection separation distance was 3 cm also for Fp2 acquisitions.

**Figure 2:**
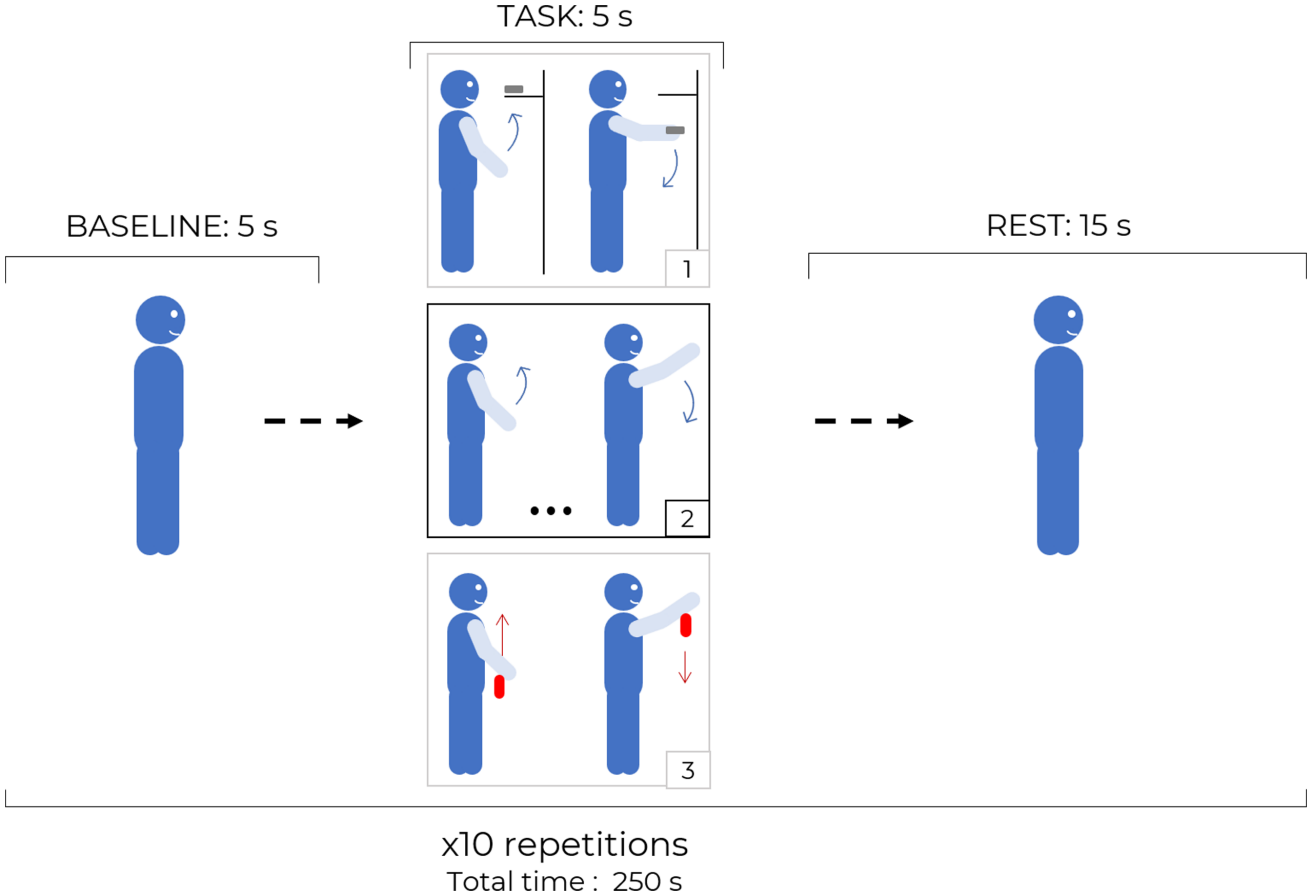
Protocol structure: each block is composed by 5 s baseline, 5 s task and 15 s rest periods and it is repeated 10 times for the same task. The three different tasks are sketched in the black and gray squares: 1. ARG, 2. AR, 3. HAR, red arrows indicate passive movements while blue arrows indicate active and independent motion of the subject.

For each task, the subject’s arm moved (actively or passively) from the resting position, longitudinally to the body, up to the height of the eyes, keeping the elbow straight and returning to the resting position. Each block consisted of 5 s of baseline, 5 s of task and 15 s of rest, repeated 10 times. The synchronization of the tasks was performed using audio commands. In the arm raising task (AR, not goal-oriented) the subject was asked to lift his arm autonomously and lower it down in a maximum time range of 5 s. The arm raising and grasping task (ARG, goal-oriented) consisted in raising the arm to the same height of the previous exercise and grasping a small and lightweight object (20 × 50 × 10 mm). During the following repetition, the subject must reposition back the object in the original location. In the final task, helped arm raising (HAR) task, an operator was raising the subject’s arm to the height of the subject’s eye, making the subject performing a “passive” exercise, without any voluntary action by the subject. During this experiment the operator was holding the subject’s wrist for the full exercise period (baseline, task, and rest) to avoid any possible confounding effect due to the involvement of the motor cortex or SMA. If the operator perceived the subject making any active movements throughout the task, voice feedback was supplied to the subject asking to relax all arm muscles and allow the operator to solely lead the subject’s arm. A schematic of the three exercise performed during the protocol is reported in Figure 2.

### Data analysis

TD-fNIRS data were acquired as distribution of time-of-flight (DTOFs) of the photons backscattered from the probed tissue. Absolute optical coefficients have been retrieved by exploiting the convolved paths method (Zucchelli et al., 2013), which can reject superficial perturbations enhancing the contrast created by deeper layers’ variations. By computing the information about the absorption coefficient at different depths and different wavelengths it is possible to retrieve the absolute concentration of oxygenated and deoxygenated hemoglobin. By repeating this at every sampling time it is possible to calculate the hemodynamic response function (HRF).

The HRF used to fit our data is therefore composed by the sum of three gamma functions (Γ) with different signs, and different peak delays (Eq. 1) to possibly include the several different processes involved in the neurovascular coupling mechanism (e.g., feed-forward effect, fee-back effect, initial dip) (Iadecola, 2017; Kamran et al., 2018). The shape of the HRF depends on the following adaptive parameters: amplitude (*A*) and peak delay of the positive gamma function from the beginning of the task (*τ*_p_); peak delays of the negative gamma functions (*τ*_d_ and *τ*_e_) and their relative amplitudes *B* and *C* (ranging from 0 to 1) with respect to the amplitude of the positive gamma function (main peak). *A*, *B*, and *C* values have opposite signs in case of deoxy-HR (negative HRF). By convolving the curve resulting from the linear combination of the three gamma functions (*h*) with a boxcar function (*N*) as long as the length of the performed task, we obtain the initial guess of the HR: *f*(*t*) (Eq. 2). The same boxcar function (*N*) has been used for each motor task and in both measuring locations.


(1) $$h\left(t\right)=A\left(\frac{t^{\tau_{e}+\tau_{p}}\;e^{-t}}{\Gamma \left(\tau_{e}+\tau_{p}+1\right)}-B\frac{t^{\tau_{e}+\tau_{p}+\tau_{d}}\;e^{-t}}{\Gamma \left(\tau_{e}+\tau_{p}+\tau_{d}+1\right)}-C\frac{t^{\tau_{e}}\;e^{-t}}{\Gamma \left(\tau_{e}+1\right)}\right)$$

(2) $$f\left(t\right)=h\left(\tau_{p},t\right)*N$$

The initial HRF will then be fitted through the Levenberg–Marquardt algorithm to minimize the mean square error between the theoretical HRF and the measured data. The fitted parameters are: *A*, *B*, *C*, *τ*_p_, *τ*_d_, and *τ*_e_. Boundaries and initial guesses for the fitting process are summarized in Table 1. Note that since the “initial dip” (*B*) and the “undershoot” (*C*) amplitudes are relative to the main peak amplitude (*A*), their values range from 0 to 1. Their, their initial guesses were set to 0.5. The fitting results were examined using Pearson’s correlation coefficient (*R*) to determine the quality of the fitting procedure: *R* < 0.3 translates in weak correlation, 0.3 < *R* < 0.7 in moderate correlation and *R* > 0.7 in high correlation.

**Table 1:** Boundaries (Max and Min) values for the fitted parameters and their initial guess value.

	*A* (μM)	*τ*_e_ (s)	*τ*_p_ (s)	*τ*_d_ (s)
Min	0 (O_2_Hb)–5 (HHb)	1	4	7
Max	5 (O_2_Hb)–0 (HHb)	6	14	19
Initial guess	1 (O_2_Hb)–1 (HHb)	5	7	12

Average results obtained from the HRF fitting procedure over the entire population have been analysed individually, to look for significant differences between average values within the three different tasks. For each comparison, a paired two-sample, two-tailed *t*-test has been exploited. The null hypothesis was formulated assuming that there is no significant difference between values being compared. Additionally, the Kolmogorov–Smirnov (KS) test of normality with KS *p*-value >0.05 was used to make sure that the samples under study did not differ significantly from a normal distribution.

The two measuring channels (pre-frontal cortex and motor cortex) have been treated independently. Since no significant activations have been recorded in the prefrontal cortex (control channel), statistical analysis on the hemodynamic responses have been focused on the motor cortex channel only. To address the issue of multiple comparisons due to the multiple *t*-tests performed on the same population performing different tasks, a correction for multiple comparisons was applied using the Bonferroni method, lowering the potential occurrence of false positives. Considering the comparisons within three distinct tasks, a total of three comparisons were considered in the analysis. This correction resulted in an adjusted significance threshold of alpha = 0.017, reducing the likelihood of Type 1 errors (false positives). Therefore, to determine statistical significance, differences with *p*-values below the threshold *p* = 0.017 were deemed statistically significant.

## Results

**Figure 3:**
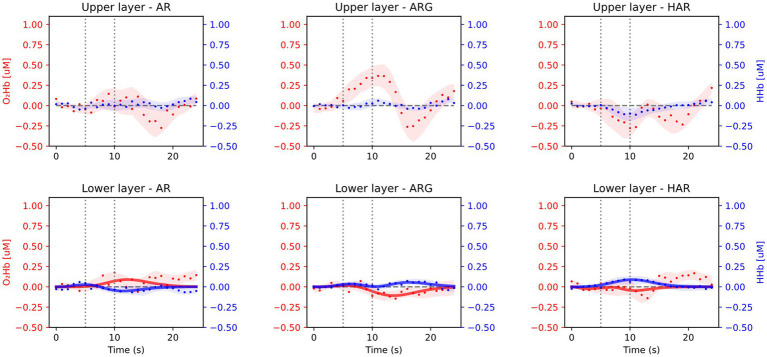
Average results from the prefrontal cortex acquisition during the three motor tasks: arm raising (AR), arm raising and grasping (ARG), and helped arm raising (HAR). Shaded areas represent the standard error calculated across all subjects. Upper row shows results from the extra-cerebral layers and lower row from cerebral cortex.

**Figure 4:**
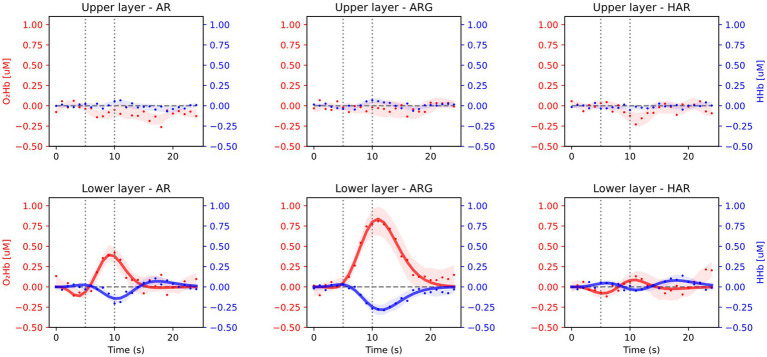
Average results from the motor cortex position during the three motor tasks: arm raising (AR), arm raising and grasping (ARG), and helped arm raising (HAR). Shaded areas represent the standard error calculated across all subjects. Upper row shows results from the extra-cerebral layers and lower row from cerebral cortex.

Averaged results across all 16 subjects and task repetitions are presented in Figure 3, for the prefrontal cortex (Fp2/1 position) and in Figure 4 for the motor cortex area (C3/4 position). For every exercise, results are shown in terms of hemodynamic variations from baseline values. Both figures report results relative to the upper layer, extra cerebral contribution (first row), and the lower layer, relative to the cerebral cortex (second row). Each column, from left to right, is relative to one exercise: arm raising (AR) arm raising and grasping (ARG) and helped harm raising (HAR). The adaptive HRF model used was the three-gamma-functions model, suitable to fit the initial dip of the O_2_Hb and the initial raising peak of the HHb. Only the results relative to the lower layers of the brain cortex have been fitted with the HRF model.

As expected, no significant task-related hemodynamic activations (i.e., increase in oxy-and simultaneous decrease in deoxy-hemoglobin concentrations during the task period) were noticed in the prefrontal cortex position neither in deeper cerebral layers nor in extracerebral regions, as shown in Figure 3. On the other hand, substantial brain activation was clearly visible in deeper layers of the primary motor cortex and SMA in both AR and ARG tasks, as shown in Figure 4. In these tasks, the hemodynamic response is characterized by an increase of the oxy-hemoglobin concentration and a concomitant decrease of the deoxy-hemoglobin. No systemic superficial hemodynamic variations have been noticed on average in the motor cortex area. Both oxygenated and deoxygenated hemoglobin variations during the goal-oriented task (ARG) are substantially more intense than the variations of the not goal-oriented task (AR): as a matter of fact, absolute peaks values are doubled for both hemoglobin concentrations in the ARG case.

The adaptive HRF model has been applied both on oxy-and deoxy-hemoglobin readings on every subject and every task, after averaging the five repetitions. With the aim of finding a standardized procedure to quantify the average brain activation for the three different tasks the output parameters of the fitting procedures have been averaged across all subjects and the relative differences have been studied. A deeper analysis of the HRF fitted parameters regarding the motor cortex (lower layers) area is here shown (Tables 2, 3).

**Table 2:** Average values of the fitted parameters of the HR, relative to oxygenated and deoxygenated hemoglobin concentrations over all subjects and all tasks. *B* and *C* values are unitless since they are fractions of *A*. Squared brackets signs indicates the couple of values which showed significant difference from each other (*p* < 0.017) based on the two-tailed paired *t*-test and corrected for multiple comparisons (Bonferroni method), *p*-value is reported in the text boxes next to the bracket sign. For *A* parameter, differences between oxy and deoxy values are always significant (*p* < 0.0001) but are not signed for a clearer presentation. *R* is average Pearson correlation coefficient between the measurement and the fitted HR.

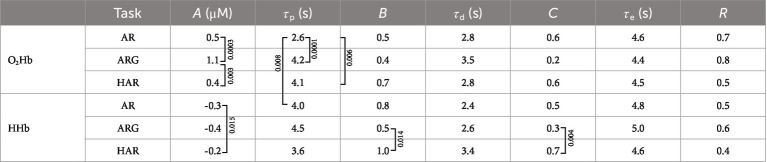

**Table 3:** Standard error of the values reported in Table 2.

	Task	*A* (μM)	*τ*_p_(s)	*B*	*τ*_d_(s)	*C*	*τ*_e_(s)	*R*
O_2_Hb	AR	0.09	0.37	0.09	0.70	0.15	0.17	0.10
	ARG	0.17	0.22	0.13	0.78	0.22	0.07	0.06
	HAR	0.07	0.35	0.15	0.73	0.20	0.17	0.08
HHb	AR	0.03	0.35	0.13	0.65	0.12	0.24	0.10
	ARG	0.07	0.25	0.09	0.76	0.08	0.09	0.09
	HAR	0.03	0.32	0.13	0.70	0.14	0.27	0.09

From Table 2 it is possible to appreciate the average values of the fitted parameters and in which cases their variations can be considered as statistically significant between different tasks. Standard errors relative to the average values are summarized in Table 3. The significance of the test has been performed exploiting the two-tailed paired two-sample *t*-test, supposing equal variances of the two populations and correcting for multiple comparison trough the Bonferroni method. *t*-test resulting in *p*-values <0.017 have been considered significant and the null hypothesis (no difference between variables under test) can therefore be rejected. Oxy-HR (oxygenated hemoglobin hemodynamic response) activations show significantly higher intensity in case of the ARG exercises compared to both AR and HAR. No significant differences have been found between the activation intensity of AR and HAR oxy-hemoglobin responses; this might be due to higher standard deviation characterizing these two values. In the AR task the activation peak of the oxygenated hemoglobin showed to occur significantly before if compared to the ARG and the HAR task. Additionally, the amplitude ratio of the undershoot of the oxy-HR activation (*B*) showed to be greater in the HAR exercise compared to the ARG one.

The significant differences found across different exercises in the deoxy-HR fitted parameters are related to the amplitude of the activation peak, the initial dip, main peak and also the undershoot, while no significant differences have been found within peaks’ delay. In particular, the amplitude of the peak (*A*) is significantly more pronounced, (larger negative value) in case of AR exercise compared to the HAR one, similarly amplitudes *B* and *C* are greater in HAR with respect to ARG deoxy-HR.

Finally, comparing oxy-and deoxy-HR responses, it has been noticed that the delay of the main activation peak (*τ*_p_) is significantly greater in the HHb compared to the O_2_Hb only in case of the AR task. No significant (inter-hemoglobin) differences have been found on other HR parameters.

## Discussion

Specific brain cortex areas can activate due to external stimulus or voluntary actions. Brain activation related to a particular task performed by a subject will be defined as “functional” brain activation. During the stimulus, a functional cerebral hemodynamic response typically involves a rise in oxygenated hemoglobin concentration with a concurrent reduction (generally with a lower intensity) in deoxygenated hemoglobin concentration, followed by a relaxation trend toward the original values. The cerebral hemodynamic response in the brain cortex area, where neuronal activation occurs, is driven by the neurovascular coupling that connects neuron and vascular functions. Relying upon the most accredited theories, this mechanism is regulated by two main phenomena: a feed-forward effect, and a feed-back effect (Kamran et al., 2018).

These two effects will eventually translate into the neurovascular response that we record via TD-fNIRS. Scientists working with the blood oxygen level-dependent (BOLD) signal from functional magnetic resonance imaging (fMRI) have established models to characterize such hemodynamic responses (Boynton et al., 1996; Handwerker et al., 2004). Recent publications presented an adaptive HRF model which can be used to fit also fNIRS acquisitions (initially retrieved by CW-fNIRS measurements only), as presented by Uga et al. (2014). The HRF in the mentioned work is composed by the linear combination of two gamma functions, having opposite signs and fixed delay between their peak positions.

It has also been reported that HRF can show different characteristics in different brain regions (Jasdzewski et al., 2003; Kamran et al., 2015). Furthermore, it is well-known that the hemodynamic signal has inter-subject variability as well as inter-trial variations. A modification of the previous model can be proposed by adding one third gamma function having negative sign, in the timeframe between the beginning of the task and the peak of the first gamma function [an example is presented in (Kamran et al., 2018)]. This additional variable allows to account for a faster process of the cerebral hemodynamic response, often called “initial dip” (Kamran et al., 2018), which is most probably related to the feed-forward mechanism of the neurovascular coupling. This approach is not new in the fNIRS world but, to our knowledge, it is the first time it has been applied to TD-fNIRS data. From the results presented in the previous section, on average, the fitting of the hemodynamic measurement with the adaptive HRF model here proposed, showed moderate *R* > 0.3 to strong *R* > 0.7 correlation (Table 2 reporting average values and *p*-values form the *t*-test and Table 3 reporting their standard errors). Only three subjects showed an average correlation (*R*) over the three tasks lower than 0.3 related only to the deoxy-HR fit. All subjects showed more than 0.3 average correlation, and more than half of the population showed average correlation (over hemoglobins and tasks) greater than 0.7.

In Table 2, the stronger activation seen in the ARG task can be explained by more intense planning of the movement and higher complexity of the overall task. From Figure 4, we also notice that, on average, the peak of the activation of the ARG task is delayed with respect to the AR task for both HHb and O_2_Hb (significant only in case of oxy-HR *p*-value = 0.0001, Table 2), this could be potentially related to the more complex planning and execution of the task, requiring more time to be processed. On the other hand, in the helped arm raising (assisted) task, we did not notice any substantial activation in the motor cortex. Given the passive nature of the exercise (in its optimal execution, where the subject does not plan/perform any movement) no brain activation is expected to occur. Only slight activations were seen on a few subjects, with very low intensity, mainly related to oxygenated hemoglobin variation. It is important to notice that fitting parameters outputs resulting from AR and in particular HAR tasks are highly variable, mainly due to a lower signal to noise ratio of the activations compared to ARG. Since some individuals’ HR processes are hard to be detect, average results related to the first and last peak of the HR are of lower significance. It is therefore useful to weight the relevance of the fitted parameters of a specific HRF on its main peak amplitude (*A*).

Considering this study’s limitations, it is important to acknowledge that the small number of channels employed (i.e., measurement locations) may result in a partial understanding of the overall hemodynamics of the cerebral cortex. Specifically, utilizing only two measurement locations enabled to capture hemodynamic variations solely from the prefrontal cortex and the motor cortex, while other regions of the cerebral cortex might be potentially involved in the execution of the tasks. Furthermore, the statistical analysis employed in this study is preliminary and tailored to suit the exploratory nature of this pilot investigation. To enhance significance and reliability of the presented findings, future studies should involve a larger cohort of patients.

From the literature it can be seen that most of the available studies in which similar tasks have been investigated are based on continuous wave (CW) fNIRS (Kashou et al., 2016; Jang et al., 2017). Concerning the simple arm-raising exercise, a similar task was found in literature, presented by Jang et al. (2017). In that work, 10 subjects were asked to perform three repetitions of bilateral arm raising while seated on a chair, 10 repetitions for a total task time of 20 s. The protocol included periods of 20 s of baseline before the task and 20 s of recovery after the task (rest period). A 49-channel CW-NIRS device was used, and the motor cortex was the region of interest during the analysis. The study highlighted a significant activation of the supplementary motor area and primary motor cortex zones. However, the reported conclusions only consider the values of changes in oxygenated hemoglobin and total hemoglobin (the latter can be considered a partial index of the regional blood flow) without including deoxygenated hemoglobin information. This weakens the reliability of the exposed results by not providing a comprehensive hemodynamic perspective of the functional response, but it does assist us in better identifying the brain area that will be most engaged in the specified motor task.

In the case of goal-oriented task (arm raising and grasping), no previous studies were found that proposed similar protocols, but it is possible to estimate the extent of the related brain activation by combining information from arm raising and grasping exercises. Grasping tasks are often studied individually and different objects might be selected to be grasped. From the work of Kashou et al. (2016) we foresee that the motor cortex activation related to a grasping task will have a comparable behavior to a finger-tapping exercise. Given the higher complexity of the overall movement, we expect the goal-oriented activation to be more intense with respect to the non-goal-oriented one. Healthy subjects brain activation related to finger-tapping task have already been explored by the authors with the same TD-fNIRS device exploited in this study (Lacerenza et al., 2020).

Since in previous literature no studies have been found comparing passive motor task (e.g., helped arm raising) with active motor task (e.g., standard arm raising task), in this work, the helped arm raising task was added to the protocol to explore the possible cerebral and extracerebral hemodynamic triggered by a passive action. Given that, the motor cortex area will not be actively involved, no significant motor cortex activation was expected in healthy subjects.

The evaluation of brain activation during assessment phase and training phase is of high interest in the neurorehabilitation field. The work by Maier et al. (2019) unifies the neuroscientific literature relevant to the recovery process and rehabilitation practice in order to provide a synthesis of the principles that constitute an effective neurorehabilitation approach. The authors investigate on how to implement the knowledge of these principles and formalize rehabilitative protocols that truly modify the neural substrate and promote skills not only in stroke patients but also in other neurodegenerative diseases such as Parkinson’s disease. One of the most important principle is the goal-oriented therapy. As in the article of Maier et al. this work confirms the importance in brain activity of goal-oriented tasks compared to non-goal-oriented ones.

The supplementary motor area, which was considered in this study is involved in important clinical conditions. This area has come under increasing scrutiny from cognitive neuroscientists, motor physiologists and clinicians given its crucial role in linking cognition to action (Nachev et al., 2008). The work by Jacobs et al. (2009) shows that the supplementary motor area contributes to the timing of the anticipatory postural adjustment and that participants with Parkinson’s disease exhibit impaired timing of their anticipatory postural adjustments, in part, due to progressive dysfunction of circuits associated with the supplementary motor area. TD-fNIRS devices could investigate the activation of this crucial area during the execution of even more complex functional movements, such as direction changes in gait, which are really complex and difficult for patients with Parkinson’s disease (Park et al., 2020).

The investigation using TD-fNIRS could also be fundamental to study and implement other neurorehabilitation principles, such as practice characterized by a particular task. Practice for a task requires that the conditions for performing a task (neuro-muscular synergies) are selective and specific for that same task both in terms of sensorimotor performance and contextual factors. Specialized training facilitates motor learning even during the retention phase, and reduces inhibition of the non-affected hemisphere on the affected one and the relationship between them (Maier et al., 2019). The work from Walsh et al. (2008) using fMRI and Structural Equation Modelling (SEM) to compare unimanual and bimanual movements shows that the dominant hemisphere appears to initiate activity responsible for bimanual movement, and that the networks involved in producing unimanual movements are distinct from the networks involved in contralateral unimanual movements. These findings provide a better understanding of cortical motor physiology in both healthy individuals and those with neurological damage.

Due to their portability, ease of use, and capability to track in real time the hemodynamic activity of key cerebral regions, TD-fNIRS devices may be used to monitor brain activity during functional movement, functional bimanual exercises, or in ongoingmovement.

TD-fNIRS has already been used to record prefrontal region activity during cognitive activities. As the prefrontal cortex is crucial in processing the cognitive, emotional, and behavioural information required for normal functioning in daily life, interest in this topic has grown. Dysexecutive disorders that may occur following damage to the prefrontal cortex can include difficulties in planning and organization, working memory, mental flexibility, impulse control, and emotional regulation (Jones and Graff-Radford, 2021). These disorders can negatively affect the individual’s ability to perform work activities and to interact effectively with others in a social environment. For example, difficulties in planning and organization can make difficult for an individual to perform complex work tasks or manage his daily life (Diamond and Ling, 2019). Because executive function networks encompass several circuits of cortico-subcortical brain regions and cerebellar sites, the exploitation of TD-fNIRS is promising also in cognitive rehabilitation environments to assess and enhance brain activation (trough tailored exercises) in specific areas associated with executive function.

## Conclusion

Brain activations from 16 healthy subjects have been successfully recorded during upper limb motor exercises, exploiting compact commercial TD-fNIRS devices, without any restriction on subject enrollment due to hair/scalp characteristics. Quantitative oxygenated and deoxygenated hemoglobin regional concentrations have been recorded, probing extracerebral and cerebral tissues, and in two different brain locations simultaneously. Goal-oriented, not goal-oriented and passive tasks have been explored and consistent results have been retrieved across subjects. Thanks to the employed HRF model, it was possible to quantitatively compare shape, amplitude and timing of the motor cortex HRs in the different tasks. The active arm raising and grasping task (ARG) resulted in a significantly stronger activation compared to the active arm raising task (AR), while, on average, the passive arm raising task did not produce any substantial activation in the brain cortex. The valuable results here reported are representative of upper limb motor activations in healthy subjects, forming an explorative dataset to be compared in the future with brain activations in pathological conditions.

This study based on measurements on heathy subjects, supports that the use of TD-fNIRS in the field of neurorehabilitation has the potential to monitor motor learning in individuals with neuromuscular impairments by providing a quantitative and non-invasive method for measuring changes in brain during motor exercises. This can be used to design rehabilitation programs that target specific brain regions, potentially optimizing motor learning and following progress over time.

## Data availability statement

The raw data supporting the conclusions of this article will be made available by the authors, without undue reservation.

## Ethics statement

The studies involving human participants were reviewed and approved by ethics commitee of Politecnico di Milano Milano, Italy. The patients/participants provided their written informed consent to participate in this study.

## Author contributions

All authors contributed to the article and approved the submitted version.

## Funding

This work has received funding from the European Union’s Horizon 2020 research and innovation programme under grant agreements No.101016087 (VASCOVID).

## Conflict of interest

ML, MB, DC, and AT are co-founders of PIONIRS s.r.l., Italy.

The remaining authors declare that the research was conducted in the absence of any commercial or financial relationships that could be construed as a potential conflict of interest.

## Publisher’s note

All claims expressed in this article are solely those of the authors and do not necessarily represent those of their affiliated organizations, or those of the publisher, the editors and the reviewers. Any product that may be evaluated in this article, or claim that may be made by its manufacturer, is not guaranteed or endorsed by the publisher.
